# Coupled Socio-Environmental Changes Triggered Indigenous Aymara Depopulation of the Semiarid Andes of Tarapacá-Chile during the Late 19th-20th Centuries

**DOI:** 10.1371/journal.pone.0160580

**Published:** 2016-08-25

**Authors:** Mauricio Lima, Duncan A. Christie, M. Calogero Santoro, Claudio Latorre

**Affiliations:** 1 Departamento de Ecología, Facultad de Ciencias Biológicas, Pontificia Universidad Católica de Chile, Santiago, Chile; 2 Center of Applied Ecology and Sustainability (CAPES), Santiago, Chile; 3 Laboratorio Internacional de Cambio Global (CSIC-PUC), Santiago, Chile; 4 Laboratorio de Dendrocronología y Cambio Global, Instituto de Conservación, Biodiversidad y Territorio, Universidad Austral de Chile, Valdivia, Chile; 5 Center for Climate and Resilience Research (CR)2, Santiago, Chile; 6 Laboratorio de Arqueología y Paleoambiente, Instituto de Alta de Investigación, Universidad de Tarapacá, Arica, Chile; 7 Centro del Desierto de Atacama, Pontificia Universidad Católica de Chile, Santiago, Chile; 8 Institute of Ecology & Biodiversity (IEB), Santiago, Chile; University of Waterloo, CANADA

## Abstract

Socio-economic and environmental changes are well known causes of demographic collapse of agrarian cultures. The collapse of human societies is a complex phenomenon where historical and cultural dimensions play a key role, and they may interact with the environmental context. However, the importance of the interaction between socio-economic and climatic factors in explaining possible breakdowns in Native American societies has been poorly explored. The aim of this study is to test the role of socio-economic causes and rainfall variability in the collapse suffered by the Aymara people of the semiarid Andean region of Tarapacá during the period 1820–1970. Our motivation is to demonstrate that simple population dynamic models can be helpful in understanding the causes and relative importance of population changes in Andean agro-pastoral societies in responses to socio-environmental variability. Simple logistic models that combine the effects of external socio-economic causes and past rainfall variability (inferred from Gross Domestic Product [GDP] and tree-rings, respectively) were quite accurate in predicting the sustained population decline of the Aymara people. Our results suggest that the depopulation in the semiarid Tarapacá province was caused by the interaction among external socio-economic pressures given by the economic growth of the lowlands and demands for labor coupled with a persistent decline in rainfall. This study constitutes an example of how applied ecological knowledge, in particular the application of the logistic equation and theories pertaining to nonlinear population dynamics and exogenous perturbations, can be used to better understand major demographic changes in human societies.

## Introduction

Socio-economic and environmental changes are well known causes of demographic collapse of agrarian cultures [1, 2]. Yet, the interplay between these drivers is often complex and can be a challenge to model in the light of future predicted climate change [3]. More often than not, greater understanding of the relevant factors involved can be resolved by increasing our understanding of how the interaction among socio-economic and environmental factors bring about changes in population and institutional structures in pre-industrial societies [4].

A growing body of literature relates the population decline and collapse of pre-industrial civilizations to socio-environmental changes as the most important factors that may be triggered these processes [2, 5]. For example, past climatic changes related to unusual cooling periods appear to be the cause of the major demographic and economic crisis in pre-industrial Europe and China during the last millennia [6, 7], and a severe drying trend may triggered the population collapse of the Maya political system [8, 9]. The expansion and decline of societies can be tightly related to the long term variability in food sources by expansion-contraction cycles in agricultural productivity [10, 11]. Clearly, decadal to centennial scale climatic anomalies can be an important factor for these population fluctuations, yet the collapse of human societies is a complex phenomenon where historical and cultural dimensions play an important role, and they may interact positively with the environmental context.

A case in point are Native Americans, which have experienced several major population declines, some of them related to environmental drivers, such as those that occurred throughout the mid to late Holocene [12, 13]. An often cited example is the decline of the Anasazi civilization in the US southwest which has been attributed to a series of severe droughts that occurred during the Medieval Climate Anomaly [14, 15]. Another well-studied (yet controversial) case is the collapse of the Maya civilization in response to long-term droughts and overpopulation in the southern Maya lowlands of Mesoamerica during the Little Ice Age [8–11]. In fact, other studies have argued that the collapse of Maya society has to be viewed as the result of different causal factors operating at different centuries and areas [16–18]. Nevertheless, the cultural and productive system of European colonization during the sixteenth century generated a massive, widespread depopulation of almost all Native American peoples through epidemics, slavery, war and genocide [19]. A controversial case is the collapse of the Easter Island population, while studies support the view of combined socio-political and environmental drivers [20–23], others propose that massive deforestation was driven by the interaction of human population and rats, but the demographic and cultural collapse occurred after the arrival of Europeans [24, 25]. Therefore, the role of the interaction between socio-economic and climatic factors in explaining possible breakdowns in Native American societies has been poorly explored beyond the case of the Maya society [17, 18, 26].

**Fig 1:**
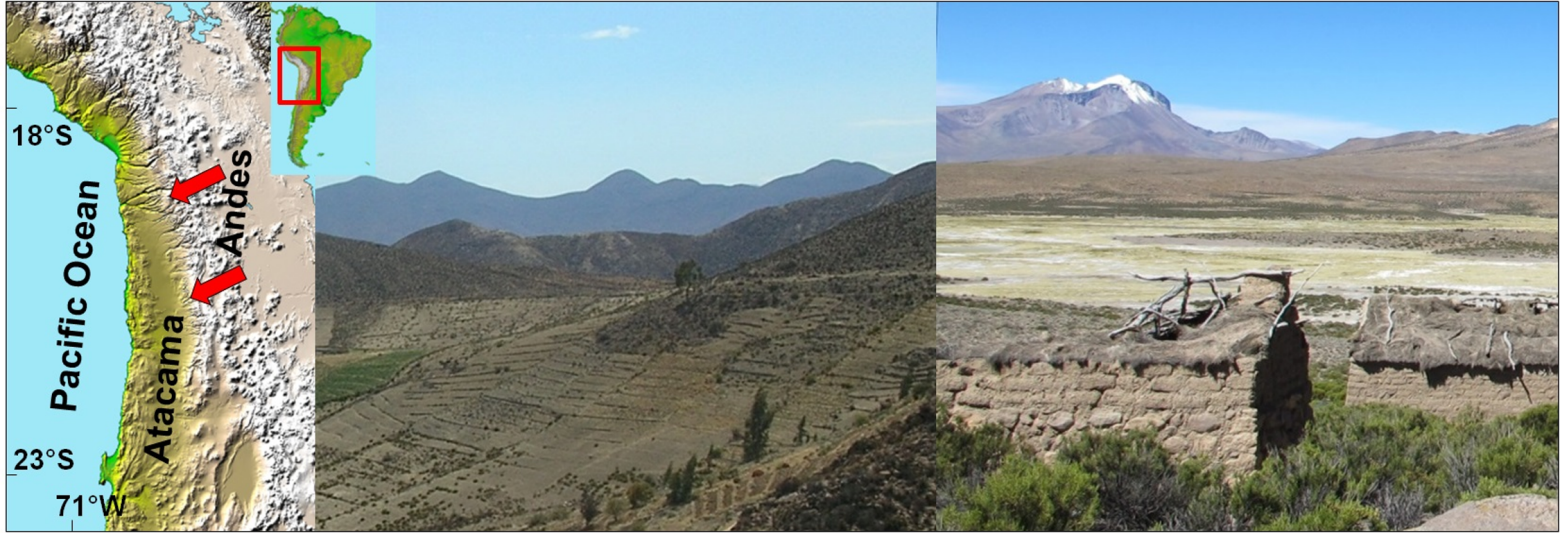
Scheme of the Aymara population flow from the Andes of Tarapacá to the Atacama lowlands. Scheme of the Aymara population flow from the Andes of Tarapacá to the Atacama lowlands represented by the red arrows (left), and examples of abandonment of agricultural terraces (center) and houses (right) by the Aymara people in the Andes of Tarapacá. Original shaded relief image (left) obtained from NASA/JPL/NIMA.

The semiarid Andes of Tarapacá are located between the Atacama Desert and the Altiplano. The region has been subject to large variations in rainfall patterns throughout the late Quaternary [27, 28]. For example, a recent study examined the consequences of rainfall variability during the last 2500 years in the hyperarid core of the Atacama Desert, where hydraulic societies (e.g. as originally described by Santoro et al. [29] flourished during brief (several centuries) interludes of augmented water availability caused by increased rainfall in the Andes [30]. The Spanish domination, however, and subsequent development of mining and collapse of native social networks in the sixteenth century caused a massive collapse of the native populations of the Andes of Atacama [31], which then recovered during the eighteenth and early 19th century [32]. This pattern is well known to the Aymara people of the province of Tarapacá, an agro-pastoral society that exhibited an important population recovery from 1650 to 1830 AD [32]. However, during the second half of the 19th and early 20th century, the Aymara of Tarapacá suffered a drastic population decline termed the “Aymara Holocaust” [32]. The proposed causes for this population decline are basically of socio-economic nature and closely related to the migration waves of this agro-pastoral society from the highlands of Tarapacá to the coastal cities and the nitrate mines of the Atacama lowlands [32] (Fig 1).

Moreover, tree-ring based precipitation reconstructions and rodent midden records from the Altiplano show that a major pluvial phase during the first half of the 19th century was followed by a persistent decrease in rainfall since the second half of the 19th century [28, 33]. This prominent decrease in rainfall could clearly have interacted with socio-economic drivers in triggering the decline of the agro-pastoral Aymara. Yet to date, no study has addressed this possibility.

The aim of this study is to test the role of socio-economic causes and rainfall variability in the collapse suffered by the Aymara people of the Tarapacá province during the period 1820–1960. Our motivation is to demonstrate that simple population dynamic models can be helpful in understanding the causes of population changes in response to socio-environmental variability. We have applied these models for deciphering the possible interaction between external socio-economic factors and rainfall variability in explaining the Aymara depopulation.

## Methods

### Tree-ring data as a proxy of precipitation

The tree species *Polylepis tarapacana* is ubiquitous in the semiarid Andes of Tarapacá and with its exactly-dated annual resolution and the demonstrated sensitivity of radial growth to moisture variations, represents an excellent proxy for past precipitation variability and plant productivity in the South American Altiplano [34–37]. In this study, we developed a *P*. *tarapacana* tree-ring chronology as a proxy of past precipitation by sampling a population from the Andes of Tarapacá (Queñiza site; 19°22`S, 68°55`W; 4303 m a.s.l.). We acknowledge the Chilean Forest Service CONAF for local support and permission to collect tree-ring samples, and the national water agencies DGA-Chile and SENAMHI-Bolivia for providing the instrumental precipitation records.

**Fig 2:**
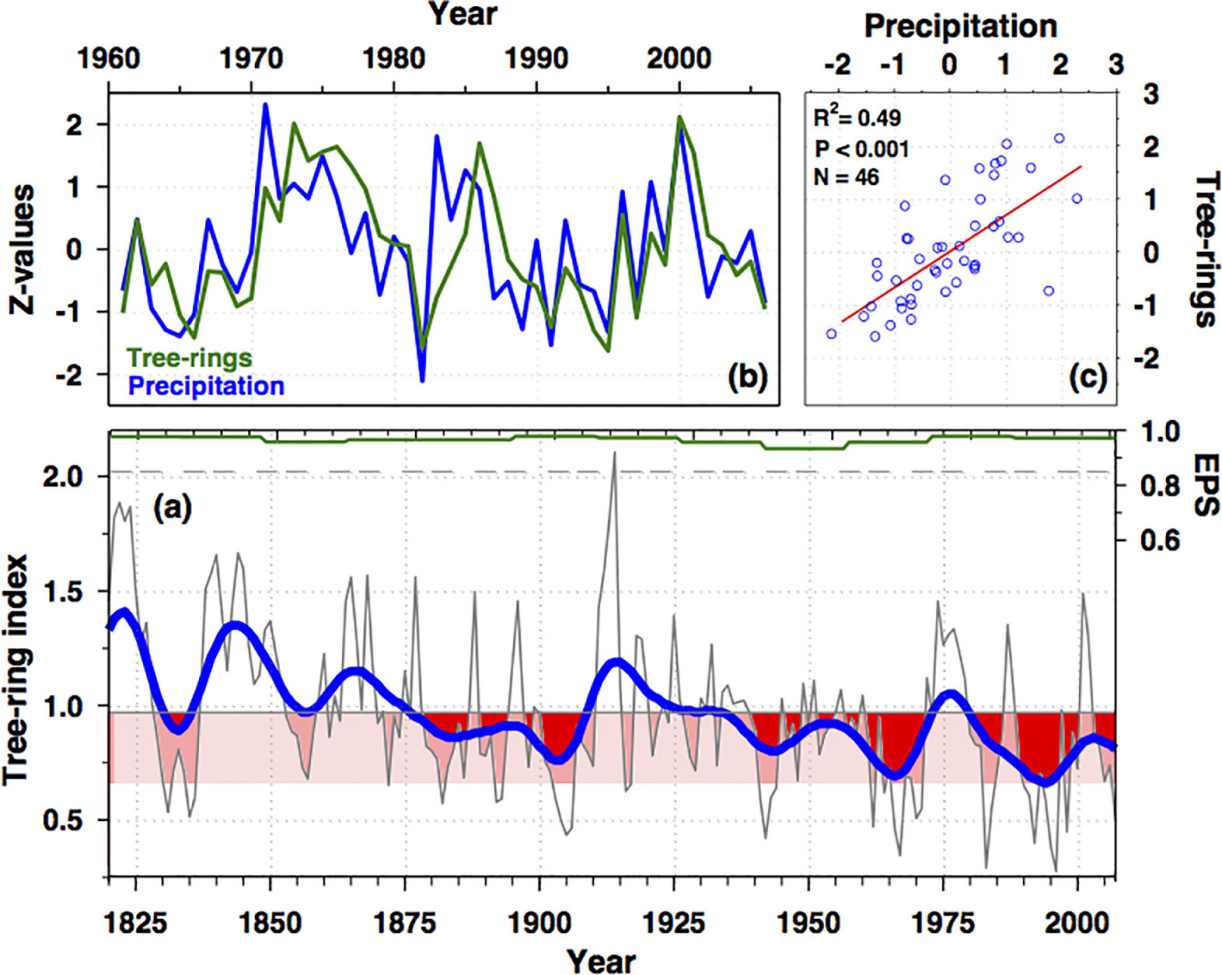
The *Polylepis tarapacana* tree-ring chronology from the semiarid Andes of Tarapacá as a proxy of past precipitation. (a) The tree-ring chronology from 1820 to 2007. To emphasize the low-frequency variations a 35-year low-pass filter (gaussian) of the chronology is shown in blue, and the red shaded denotes the low- frequency area below the 1820–2007 mean. The upper green line denotes the Expressed Population Signal (EPS) statistic calculated for 30-year window with an overlap of 15, and the light grey dashed line the 0.85 EPS value. (b) Comparison of the instrumental regional record of precipitation and the tree-ring chronology time series for their common period. (c) Scater plot and linnear regression of regional precipitation versus tree-ring indices. For (b) and (c), the tree-ring chronology and precipitation values are expressed as normalized values for their common 1961–2006 period. The regional precipitation record was conformed by the following stations: Putre 18°11`S, 69°33`W, 3545 m a.s.l.; Parinacota 18°12`S, 69°16`W, 4420 m a.s.l.; Visviri 17°37`S, 69°28`W, 4080 m a.s.l.; Colchane 19°16`S, 68°38`W, 3700 m a.s.l.; Caquena 18°03`S, 69°12`W, 4400 m a.s.l.; Patacamaya 17°15`S, 67°57`W, 3789 m a.s.l.; and SP Lipez 21°41`S, 66°37`W, 4165 m a.s.l..

Due to the eccentric radial growth patterns of *P*. *tarapacana*, cross-sections were collected from branches of living trees and sub-fossil wood. Samples were prepared following standard dendrochronological techniques as outlined in Stokes and Smiley [38]. Tree-rings were visually cross-dated to the year of ring formation [39] and measured under a binocular stereoscope with 0.001 mm precision. We followed the Schulman [40] convention for the Southern Hemisphere, which assigns dates of annual rings to the year in which the radial growth started. The cross-dating quality of the tree-ring series was checked with the computer program COFECHA which calculates segmented time-series correlations between individual tree-ring series after emphasize its high frequency variability [41]. To develop standard tree-ring chronology individual ring-width measurements were detrended to remove variability in the time series not related to climate such as tree aging or forest disturbances. The measured ring width of year *t* was divided by the year *t* value of a fitted negative exponential curve or a linear regression. The ring-width chronology was calculated averaging the detrended tree-ring series with a bi-weight robust mean estimation by the ARSTAN40c program [42]. The variance of the chronology was stabilized using the method described by Osborn et al. [43], and the quality of the chronology was assessed by calculating the statistic Expressed Population Signal (EPS) using a 30-years window with an overlap of 15 years between adjacent windows [44]. The calculated tree-ring width chronology was composed of 51 tree-ring series, and the time period utilized in the present study present an EPS value > 0.85 and more than 30 series each year (Fig 2).

### National Economic Data as external socio-economic proxy

We used the annual data on per capita Gross Domestic Product (GDP) of Chile for the period 1820–1970 extracted from the Maddison Project data base as a proxy for the nitrate industry activity [45]. The regional economic importance of the Nitrate exploitation was so high during the late 19^th^ century that became one of the main triggers of the War of the Pacific between Chile and Peru-Bolivia [46]. It is important to note that although the nitrate mines of Tarapacá Region were in Peru until 1879, most of the export profits of such mines greatly increased the wealth of owners in Chile and the United Kingdom with subsequent impact on gross domestic product in Chile [47]. Importantly, there were no major differences in the activity of nitrate industry after the disputed territories were annexed to Chile during 1879, nor on the location and movements of the Aymara people [46, 47].

### Demographic data of Aymara society and Population model

Population data from 1650–1970 AD of the Aymara people present in the Tarapacá province (today located in northern Chile but originally in southern Peru until the Ancón Treaty, signed in 1883) were extracted from Van Kesel [32] and Chilean National Census Data (Instituto Nacional de Estadísticas; INE). The R-function represents the relationship between the human per capita growth rate over a given period of time and the human population at the beginning of that period of growth, and can be estimated as:

$$R_{t}={\ln\;N}_{t}-\ln\;N_{t-d}.$$ (1)

R can also be expressed in terms of birth and death rates:

$$R_{t}=\ln\left(1+B-D\right).$$ (2)

Where R is the realized per capita growth rate, B and D are the per capita birth and mortality rates, respectively, and d is the time period between population estimates. In this study, the time scale for the population analyses was 10 years. Although the generational time step for humans is around 30 to 25 years [48], we preferred to use an interval of 10 years in order to increase the number of data points available.

### Theoretical models of human population dynamics

The starting point was to model human populations using the exponential growth model which is a fundamental property of all population systems [49]. We used the discrete time version:

$$N_{t}=N_{t-1}∙e^{R_{m}}.$$ (3)

By defining Eq 3 in terms of the R-function using Eq 1 and log transforming the Eq 3, a model of exponential growth is obtained, where the additive effects of climate can be added:

$$R_{t}=R_{m}+g(Z_{t-d}).$$ (4)

Where *R*_*t*_ is the realized per capita growth rate estimated from data (*R*_*t*_ = *ln N*_*t*_*−ln N*_*t-1*_), *R*_*m*_ is a positive constant representing the maximum per capita growth rate, and *g* is a simple linear function (+ or -) of the exogenous forcing variables. Eq 4 represents the dynamics of a human population in a varying unlimited environment.

The alternative hypothesis is that the Aymara population was limited by resources (food, land, crop yield). We therefore used a simple model of intra-specific competition, the generalized exponential form of the discrete logistic model [50, 51]:

$$N_{t}=N_{t-1}∙e^{\left[r_{m}-c∙N_{t-1}^{a}\right]}.$$ (5)

Where *N*_*t*_ represents the population size at time *t*, *r*_*m*_ is a positive constant representing the maximum finite reproductive rate, *c* is a constant representing competition and resource depletion, and *a* indicates the effect of interference on each individual as population size increases [49]. A value of *a* >1 indicates that competition intensifies with population size, and *a* < 1 indicates that per capita competition decreases with population size. The parameter *c* represents the equilibrium population size [49]. By defining Eq 5 in terms of the Rfunction, defining *R*_*t*_ = *ln N*_*t*_*−ln N*_*t-1*_, log-transforming Eq 5, and defining the population size in natural logarithm *X*_*t*_ = *ln (N*_*t*_*)*, the Ricker [51] model of Eq 5 can be expressed as:

$$R_{t}=R_{m}-e^{\left[a∙X_{t-1}+C\right]}.$$ (6)

Where *R*_*t*_ is the realized per capita growth rate *R*_*t*_ = *log*_*e*_ *(N*_*t*_*/N*_*t-1*_*)*, *R*_*m*_ = *log*_*e*_*(r*_*m*_*)*, *a* is the same parameter as in Eq 5, *C* = *log*_*e*_*(c)*, and *X* = *log*_*e*_*(N)*. Therefore, this model can be used for representing the hypothesis that precipitation variability has an impact on food supplies through the effect on land carrying capacity in pre-industrial societies [49, 50]. The correct model in this scenario is that the carrying capacity (equilibrium point) is affected by the exogenous forcing term. In this case the climate (tree-rings) and socio-economic change shifts the R-function curve along the x-axis without changing the slope at equilibrium, representing a ‘‘lateral” perturbation in the Royama [49] framework:

$$R_{t}=R_{m}-e^{\left[a∙X_{t-1}+C+g(Z_{t-d})\right]}.$$ (7)

Models from Eqs 4 and 7 were fitted by nonlinear least squares using the nls library in the R program (R Development Core Team 2011, available at http://www.r-project.org) and ranked according to the Bayesian information criterion (BIC) or Schwarz criterion [52]. For clarity, BIC weights were also included in the results. Minimum BIC was selected to determine the best model. Finally, simulations were carried out to elucidate the capacity of the models to describe the real dynamics. Simulations were carried out only using the first real value of the time series, and then running the algorithm using each model with their estimated parameters to obtain the simulated values. We used the Pearson’s correlation coefficient between the observed and predicted numbers to assess model predictions.

## Results

**Fig 3:**
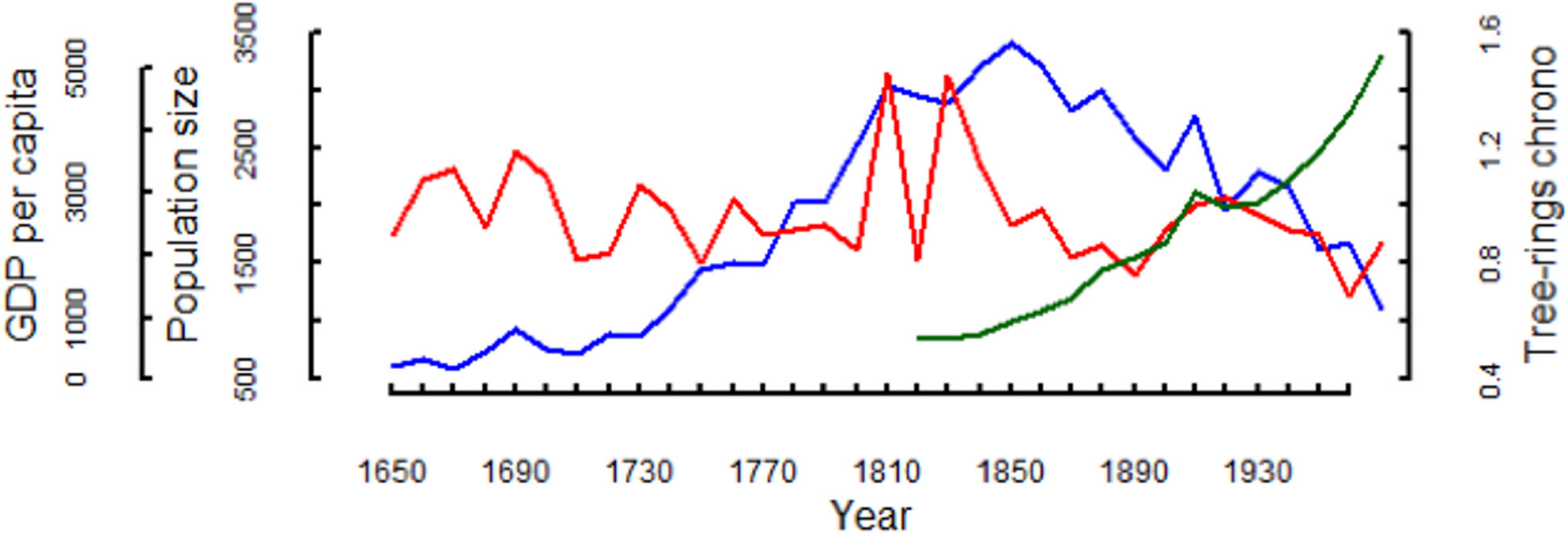
Aymara population dynamics for the period 1650–1960 at the Tarapacá province (blue line), the *P*. *tarapacana* tree-ring chronology as a proxy of past precipitation in the Andes of Tarapacá (red line) and the annual data on per capita Gross Domestic Product (GDP) of Chile for the period 1820–1960 extracted from the Maddison Project data base as a proxy for the nitrate industry activity (green line).

The Aymara population of the semiarid Andes of Tarapacá from 1650 to 1850 was characterized by a clear positive trend followed by a prominent negative phase from 1850 to 1960 (Fig 3).

The negative phase appears to be related with the massive migration to the Atacama lowlands due to the exponential growth of the large-scale nitrate industry, which demanded thousands of workers [32, 33, 53]. This may have positively interacted with the precipitation decline in the semiarid Andes of Tarapacá over the agro-pastoral Aymara (Fig 3).

The next step was to split the time series for the depopulation 1820–1960. The modeling analyses showed that a logistic model with the per capita Gross Domestic Product (GDP) as a proxy for the nitrate industry as a vertical or lateral perturbation accounts for 64–67% of the observed variation in *R* values (Table 1; models 1 and 2). The model considering the per capita GDP and the tree-ring chronology as proxy for precipitation as lateral effects increased the explained variance to 81% (Table 1; model 5). The BIC value and the BIC weight of this model, including GDP and tree-growth as lateral perturbations, indicate strong empirical support for these two factors as responsible for the Aymara decline from 1820 to 1960 (Table 1). However, an additional model in which GDP and tree-growth are included as a ratio (GDP/tree-growth) showed the strongest empirical support according to the BIC and BIC weight values, indicating that both factors would interact producing the Aymara decline in a non-linear complex manner (Table 1; model 6).

**Table 1:** Population dynamic models for the Aymara Population (1820–1960) using the exponential form of logistic growth with effects of tree-rings as a proxy of rainfall and economic growth (Royama 1992); parameter values are given in the equations. The best model was chosen by using the Bayesian Information Critera (BIC). *R*_*t+1*_ = Realized per capita growth rates, *X*_*t+1*_ = ln population size, *Rain*_*t*_ = Mean tree-rings data during the 10-year interval, *GDP*_*t*_ = per capita Gross Domestic Product, *ΔBIC* = model BIC–lowest BIC, *w*_*i*_ = BIC weigths, *r*^*2*^ = proportion of the variance explained by the model, *r* predictions = Pearson’s correlation coefficient between the observed and predicted dynamics.

Population models (period 1820–1960; depopulation phase)	Log-likelihood	BIC	*p*	ΔBIC	*w*_*i*_	r^2^
1. R_t+1_ = 1.57—exp[1.08 X_t_—8.35]– 0.0006 GDP_t_	13.32	-13.09	5	9.26	0.008	0.67
2. R_t+1_ = 0.041—exp[4.89 X_t_—45.36 + 0.002 GDP_t_]	12.77	-11.99	5	10.36	0.005	0.64
3. R_t+1_ = 1.39—exp[1.02 X_t_− 7.9]– 0.0003 GDP_t_ + 0.18 Rain_t_	13.92	-11.60	6	10.75	0.004	0.69
4. R_t+1_ = 1.76—exp[0.86 X_t_− 6.43–0.10 Rain_t_]- 0.0003 GDP_t_	13.63	-11.06	6	11.34	0.003	0.68
5. R_t+1_ = 0.16—exp[4.38 X_t_− 35.36 + 0.001 GDP_t_− 3.00 Rain_t_]	17.38	-18.51	6	3.85	0.125	0.81
**6. R**_**t+1**_ **= 0.912—exp[1.11 X**_**t**_**− 9.24 + 0.0002 [GDP**_**t**_**/ Rain**_**t**_**]**	**17.94**	**-22.35**	**5**	**0.00**	**0.855**	**0.82**

**Fig 4:**
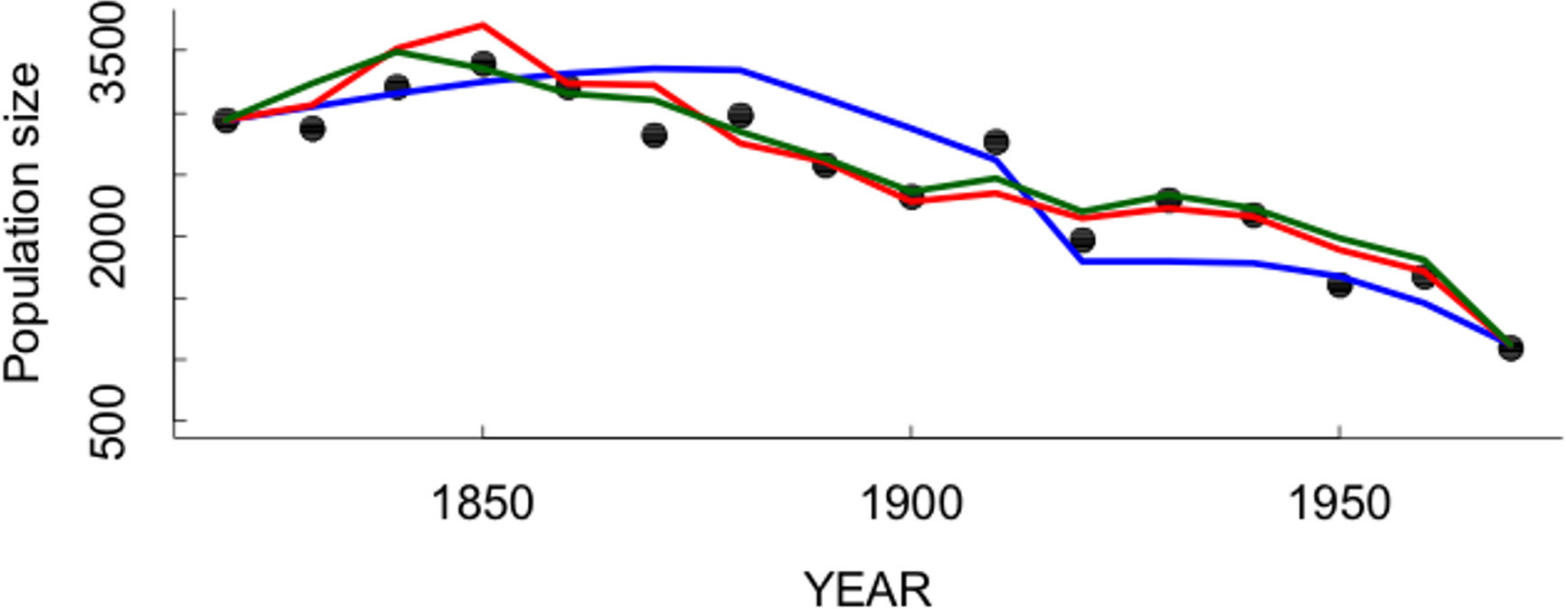
Comparison of the observed population size of the Aymara population for the period 1820–1960 (solid dots) with the predictions from models fitted to the data (Table 1). Blue line is the prediction from the logistic growth model with the additive effects of tree-rings as a proxy of rainfall, and per capita Gross Domestic Product (GDP) (model 3, Table 1). Red line is the prediction from the logistic growth model with non-additive (lateral) effects of tree-rings and per capita Gross Domestic Product (GDP) (model 5, Table 1). Green line is the prediction from the logistic growth model with non-additive (lateral) effects of the ratio between per capita Gross Domestic Product (GDP) and tree-rings (model 6, Table 1).

The simple logistic models with the effects of GDP and tree-growth were quite accurate in predicting the sustained population decline of the Aymara (Fig 4).

The simulations of the model with only the per capita GDP as a lateral perturbation seem to underestimate the decline during the 19th century and overestimate the population trend during the 20^th^ century (Fig 4; blue line). On the other hand, both models including GDP and tree-growth appear to better capture the decline trend of the Aymara population (Fig 4; lines red and green).

## Discussion

Our results suggest that the depopulation of the Aymara people in the semiarid Andean region of Tarapacá was caused by the interaction among external socio-economic pressures given by the economic growth of the lowlands and demands for workers coupled with a multi-decadal decline in rainfall. Previous studies have taken into account only the importance of the nitrate industry as a trigger of the population decline of the Aymara people [32], or just hypothesize a possible influence of the nitrate industry and precipitation [28, 34]. The present study is the first to explicitly test the role of interaction of precipitation variability and external socio-economic pressures as a factor behind this depopulation process. An interesting aspect of this study was that it could address the negative trend of the Aymara population in the Andes of Tarapacá from 1820 to 1960 through a simple population dynamics model using basic ecological principles [54]. In fact, recent studies have applied logistic models to analyze the population growth dynamics in pre-industrial China and Europe [55–57].

Our results are consistent with the described historical effects of the multinational nitrate industry and the expansion of the Chilean Republic in the Aymara population decline of the Andes of Tarapacá. The onset of the intense mining activity and increased nitrate exports to Europe during the 1830´s demanded huge amounts of labor, sparking a massive migration that in turn led in part to the decline of the local Aymara social fabric in the adjacent Andes of Tarapacá [32]. In fact, this industry was the economic engine of the region and during the early decades of the twentieth century by far, Chile’s principal export. The number of operators working in the activities of nitrate increased during the period 1880–1906 from two thousand to almost forty thousand workers [32]. The indirect effects of the nitrate industry strongly affected the agro-pastoral communities of the Andes of Tarapacá, with negative effects on the structure of the Aymara family, the agricultural labor work and production, and the political, social and economic structure of the Aymara organization. Additive to the socioeconomic factors and the acculturation process, the secular decline in rainfall that has occurred since the mid-19th century would have negatively affected the already vulnerable persistence of the agro-pastoral Aymara society in its semiarid homeland. Historically, water resources were one of the key factors driving the agro-pastoral activities of the Aymara society and its Andean predecessors [12]. It is very likely that the above-mentioned socioeconomic factors, imposed during 19th and 20th centuries by western culture, reduced the traditional resilience of the Andean Aymara society of Tarapacá to persistent drought conditions.

This article represents an attempt to show how population theory can be applied to understanding the causes of a population decline exhibited by the Aymara people of the semiarid Andes of Tarapacá. In particular, its continuous decline observed over a period of 160 years would be explained by the combined action of resource limitation, external socio-economic drivers and decreasing rainfall. In this study we explicitly demonstrate how coupled socio-environmental changes triggered the indigenous Aymara depopulation of the Semiarid Andes of Tarapacá during the late 19th-20th centuries. This research constitutes a clear example of how applied ecological knowledge, in particular the application of the logistic equation and theories pertaining to nonlinear population dynamics and exogenous perturbations can be used to better understand major demographic changes in human societies. Finally, it is important to emphasize that we are not supporting the idea of climatic determinism in the collapse of the Aymara society at Tarapacá, but that simple population dynamics principles can be useful for deciphering the complexity of human-environmental interactions. The recent Aymara collapse at Tarapacá province may help us to perceive the importance of human-environmental interactions for the challenges of global change and sustainability.
